# The Role of Urban Morphology Design on Enhancing Physical Activity and Public Health

**DOI:** 10.3390/ijerph17072359

**Published:** 2020-03-31

**Authors:** Sadegh Fathi, Hassan Sajadzadeh, Faezeh Mohammadi Sheshkal, Farshid Aram, Gergo Pinter, Imre Felde, Amir Mosavi

**Affiliations:** 1 Department of Urban Design, Bu-Ali Sina University, 94171-71946 Hamadan, Iran; sadeq.fathi70@gmail.com (S.F.); Mohamadii.faeze@gmail.com (F.M.S.); 2 Escuela Tecnica Superior de Arquitectura, Universidad Politecnica de Madrid-UPM, 28040 Madrid, Spain; Farshid.aram@alumnos.upm.es; 3 John von Neumann Faculty of Informatics, Obuda University, 1034 Budapest, Hungary; pinter.gergo@nik.uni-obuda.hu (G.P.); felde@uni-obuda.hu (I.F.); 4 Thuringian Institute of Sustainability and Climate Protection, 07743 Jena, Germany; 5 Kalman Kando Faculty of Electrical Engineering, Obuda University, 1034 Budapest, Hungary; 6 Institute of Structural Mechanics, Bauhaus Universität-Weimar, D-99423 Weimar, Germany; 7 Department of Mathematics and Informatics, J. Selye University, 94501 Komarno, Slovakia; 8 Faculty of Health, Queensland University of Technology, QLD4059 Brisbane, Australia

**Keywords:** urban morphology, physical activities, health, public health, public space, urban health, smart cities, sustainability, urbanization, urban planning, analytical network process, health economics, health informatics, Society 5.0, health information systems

## Abstract

Along with environmental pollution, urban planning has been connected to public health. The research indicates that the quality of built environments plays an important role in reducing mental disorders and overall health. The structure and shape of the city are considered as one of the factors influencing happiness and health in urban communities and the type of the daily activities of citizens. The aim of this study was to promote physical activity in the main structure of the city via urban design in a way that the main form and morphology of the city can encourage citizens to move around and have physical activity within the city. Functional, physical, cultural-social, and perceptual-visual features are regarded as the most important and effective criteria in increasing physical activities in urban spaces, based on literature review. The environmental quality of urban spaces and their role in the physical activities of citizens in urban spaces were assessed by using the questionnaire tool and analytical network process (ANP) of structural equation modeling. Further, the space syntax method was utilized to evaluate the role of the spatial integration of urban spaces on improving physical activities. Based on the results, consideration of functional diversity, spatial flexibility and integration, security, and the aesthetic and visual quality of urban spaces plays an important role in improving the physical health of citizens in urban spaces. Further, more physical activities, including motivation for walking and the sense of public health and happiness, were observed in the streets having higher linkage and space syntax indexes with their surrounding texture.

## 1. Introduction

The quality of the built environments is often recognized as one of the effective factors in reducing mental disorders in urban communities [1,2,3,4]. During the late 19th and early 20th centuries, the sudden and rapid growth of dull urban landscape and spaces, dirty streets, and rented rooms resulted in communicable diseases such as cholera and tuberculosis in the metropolitans around the world [5]. The rapid advancement of science and the expansion of cities has led to the consumption of natural resources and the growing pollution of cities [6]. Moreover, paying attention to the quality of the built environments, water supply systems, and environmental health aspects have been recognized and used as the solutions for coping with communicable diseases, as considered in large cities such as New York [7,8]. In the late 20th century, researchers in architecture and urban planning were seeking to evaluate and measure physical activities and their relationship to the built environments [9,10,11]. Each individual wishes to live in a healthy and health-oriented neighborhood and city; however, today’s neighborhoods and cities increase car dependency instead of walking [12,13]. Obviously, urban development and planning must be capable of contributing to citizens’ mental and physical health; and hence, improvement of daily activities has been recognized as a major priority in urban space design around the world [14,15].

Based on the report of Centers for Diseases Control and Prevention (CDC) risk of cardiovascular diseases and diabetes for individuals taking regular physical is minimized [16,17]. Urban open and public spaces that citizens refer to social and economic reasons are one of our most essential goals [18]. Physical activity can also result in reducing stress and anxiety and improving conditions, which is partly true for all individuals since they seek to leave the house for walking when they are feeling mentally unstable. The above-mentioned issues represent the advantages of physical activity and that the health caused by physical activity depends on the overall energy consumed during the week. The results of the study by American heart association and sports medicine institute demonstrated that individuals need 500–1000 min physical activity per week to keep their bodies in the health range [19]. Additionally, 30-min low-intensity physical activity for five days per week or at least 20 min high-intensity physical activity for three days per week is considered essential to improve the physical health of all 18 to 65-year-old individuals. Researchers believe that each time period of physical activity should at least be 10 min [20] since the relationship between physical activity and health leads to fitness, a decrease in the risk of diseases, and other advantages. Daily walking for 17 min, which is feasible by environmental design for the 25-year period, can reduce the impacts caused by cardiovascular, diabetes, obesity, and hypertension diseases to 5% [21].

Accordingly, environmental strategies are regarded as an effective tool for preventing and controlling communicable diseases during the last and recent centuries [22]. Today, environmental design is considered as a sure way to realize a healthy community despite being time-consuming. Most of the health professionals believe that urban environments should be collaboratively planned with the involvement of phycologists, architects and urban designers to react against obesity and over-weighting for encouraging physical activities [23]. Individual health is most affected by the two variables of nutrition and physical activity, and the specialized field of architecture and urbanization can play an effective role in designing and modifying the physical environment in the framework of health improvement and individual mobility [24]. The present study focused on environmental design strategies, especially the role of structure quality and spatial integration in motivating for physical activities and individual health in urban environments [25].

Based on the report of Endocrinology and Metabolism Research Institute related to Tehran University of Medical Sciences in Iran, non-communicable diseases are considered as the most important cause of mortality in Iran and the world [26]. Research connects cardiovascular diseases such as myocardial infarction, stroke, and also various types of diabetes with obesity, lack of physical activities, inappropriate food regime, and smoking, which are known as the significant factors of mortality. These diseases lead to the death of 300 individuals daily and more than 40% of mortalities based on the statistics provided by the Ministry of Health and Medical Education (MOHME) of Iran and relevant studies. In addition, myocardial infarction is regarded as the cause of more than 19% of mortalities as Iran possesses the maximum mortality rate caused by cardiac arrest in the world [27,28,29,30]. Cardiovascular diseases, stroke, diabetes, dyslipidemia, e.g., are considered as deadly diseases in Iran and today’s world. The statistics published by Ministry of Health and Medical Education of Iran (2009) and results of the studies conducted by Kelishadi [27,28] represented that around 43.9% and 16.1% of individuals are overweight and have hypertension, respectively, among which 38.9% are devoid of appropriate activity, of which a significant share is related to Hamadan city. Based on the above-mentioned statistics and information obtained from the Organization for Civil Registration of the province, the mortality rate caused by cardiovascular diseases is terrible in Iran and cities such as Hamadan. This significant issue needs comprehensive solving, although the way environmental strategies, especially designing urban structures, can be responsible for reducing these very alarming statistics in Iran, and Hamadan is regarded as the primary debate. Among all the subjects related to general health, the present study focused on physical health—particularly those diseases whose risks may be lowered by getting help from urban design as well as by increasing physical activity.

Many cities and urban neighborhoods in the contemporary era are vehicle-oriented; that is, they are designed and constructed in such a way as to provide better and faster access to vehicles. Thus, people in urban environments are not very physically active and depend on personal vehicles to a great extent. Generally speaking, compared to the previous decades, the amount of physical activity has decreased remarkably. Hence, the primary purpose of this study is to investigate the factors involved in enhancing physical activity in the main structure of the city by using the methods of urban planning. Hamadan city was selected for this study, as it is one of the metropolitan cultural-historical towns in Iran with a radial structure involving a large central square and six streets branched out with an identical angle. Previous studies have shown that the form and morphology of the city can motivate the citizens to have more mobility and physical activity in the cities. Consequently, the central question of the present study is; which criteria of urban morphology can help us to design the city in such a way to motivate the citizens to have physical presence and activity in the urban spaces. The additional question is, to what extent is each criterion quantitatively influential, and which of these criteria will have the highest priority and importance. 

## 2. Theoretical Context

### 2.1. Urban Morphology

The term Morphology is rooted in the two words of morph and logy, which means the logic of form recognition. These studies have been conducted in many fields to address physical character, structure, proportions and deformation of objects and their components [31]. This word, which was first used by the German poet Goethe and utilized in some sciences such as biology, represents a science that sought to assess the shape, form, and external structure or pattern sort based on the small Oxford dictionary (1970). Further, it is used in biology to evaluate the shape and structure of plants, animals and microorganisms, and size, shape, structure, and relationships of their components. Although it is opposed to studying the operation of organisms and their components or physiology with respect to type, their separation is artificial due to the close and reciprocal relationship between the operation and structure of organisms. The morphology and primary structure of traditional cities are designed and used based on rider and car in contrast to conventional towns [32]. In this regard, the different definitions related to urban morphology were provided as follows by considering the different approaches existing about this issue.

Urban morphology is defined as a science that assesses the generation process of the ideas and tendencies which base the form of cities [33] in order to focus on the tangible impacts of social, economic, and environmental forces [34]. Although buildings, gardens, streets, parks, and statues are always exposed to change and evolution over time, they are considered as the essential elements of morphological analyses [35,36]. In other words, the physics of the city reflects the impact and footprint of human tendencies and activities [37] As synthetic form can be related to a specific historical period, it can be caused by their designed events. The texture of a city indicates the document related to the history of its construction and the life of individuals who made and lived there [38]. Activities and requirements changed during human and community life, providing a basis for growth and variation of the physics of the city. The city as a whole, consisting of buildings and its residents, is regarded as a process and interaction between humans and their environment. In order to recognize the character of this process, studying its physical aspect is considered as the most proper basis for delineating the overall image of character since its physical nature and organization are the most tangible and sustainable aspects. Human tendencies, activities, and reactions as a part of interactions between residents and residence are regarded as relatively intangible and unsustainable.

Additionally, the use of a building may change more than its structure due to more tangibility and sustainability of its physical nature and organization. Therefore, it may provide a solid basis for starting other intangible aspects [39]. The disorderly sprawl development of cities resulted in varying the pattern related to the spatial organization of the city and its main structure. This is evident in losing the unique structure in the whole city, e.g., centralizing facilities and services in the different portions of city inappropriately and unreasonably and developing unequally in respect to socio-economic aspects. In addition, an increase in the size of cities led to the creation of physical-spatial complexities in the towns so that recognizing the main skeleton and structure of cities and its morphology based on analyzing all components of the city is regarded as almost impossible [40]. The changes in the several last centuries affected by modernism and postmodernism resulted in different cities extensively and failed to respond to a lot of their biological and social requirements according to the viewpoints of most experts. Based on the assessments conducted on the situation of cities in the end second millennium, the process of changes in city spaces was inconsistent with resident demands and requirements [41]. Table 1 represents the main elements of urban morphology with respect to the viewpoint of different theorists in this field.

**Figure 1:**
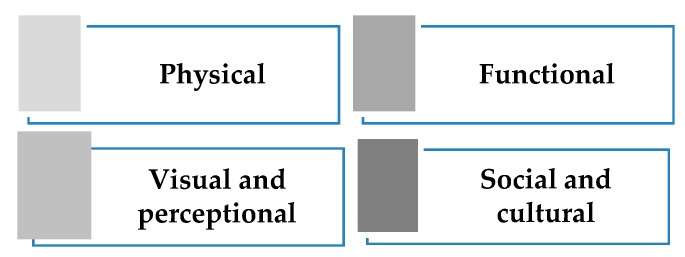
Urban morphology criteria.

Considering the mentioned theoretical basis about urban morphology, the following four criteria specified in Figure 1 can be regarded as the main criteria of urban morphology.

### 2.2. Space Syntax

The space syntax measure has been used as an indicator of street connectivity in active living research [42]. Hillier et al. first used “configuration” (pattern, order, rules and the like) to analyze the initial rules of spatial structure in 1976 [43]. Analyzing space syntax allows researchers to understand the potent relationship between form, space, and social forces [44]. Regarding this theory, the city is first divided into a discrete system including the longest visual-motor channels, in which audiences move in and perceive city structure. Next, each visual-motor channel is a criterion with a line for more advanced analyses. Then, the intersection of these lines is evaluated based on graph and mathematical analyses. The intersection of two lines represents their relationship, thus, a line having more intersection with other lines is related with higher elements in the network and is more accessible. The obtained map can be used to understand the role of each urban thoroughfare or space on extending spatial structure [45]. This method allows researchers in the field of architecture and urbanization to analyze the relationship between spatial configurations and social and behavioral structure of space and recognize, and analyze the effect of changes in urban networks on citizen mentality and behavior [43,46]. Stedman believed that space is regarded as the initial and main core of the pattern of social and behavioral events and a basis for social and cultural activities [47,48]. Spatial order is defined as the pattern of space syntax and their mutual relationship. Accordingly, each change in space syntax results in varying the amount and method of activities in spaces [49]. Space syntax is considered as an attempt on how a spatial configuration situation expresses a social or cultural meaning [50]. Predicting the amount of space used and its linkage with the daily life of people is possible based on this method. The space syntax aims to describe how human-made places such as buildings and urban space networks were formed—especially how they are articulated and aligned [50,51]. Some of the basic concepts related to this method which were used in the present study are provided as follows. 

Integration: integration or interconnection is the principal concept in the space syntax approach. An axial map is the spatial arrangement image of a city and this image can be expressed and interpreted by the integration index. The integration of a point in the map shows its relation with the overall structure. Based on this definition, if a specific space can be reached by traveling shorter spaces, that space is said to have more integration and vice versa. Connectivity: connectivity refers to the number of paths and ways that are directly linked to the path being analyzed. Depth distance: the minimum spatial distance that must be traveled from one node or path to any other node or path. The lower the depth is, the higher the integration and connectivity would be [51].

### 2.3. General Health

General health consists of the knowledge and the art of preventing diseases and maintaining and improving human health and capacity by resorting to collective work, leading to social development [52]. Simply speaking, any factor affecting an individual physically, mentally or socially, will affect his/her health and the health of other individuals in the society. Therefore, general health changes under the influence of these factors while one is performing his/her duties. Such interaction can be resulted in the flexibility of general health in different conditions and also confronting changes affecting the individuals’ and the whole society’s health [53]. Obesity: obesity can be defined as “the collapse of the physical energy equation between the amount of energy received from the environment and the amount of energy consumed in the environment”; energy can be received from the environment by taking food and calories in and it can be consumed by performing physical activity in physical environments [54]. According to numerous reports published by the World Health Organization (WHO), obesity or overweight is an important factor behind cardiovascular diseases, diabetes, high blood fat, and hypertension. According to the report published by the WHO in 2003, the rate of obesity, diabetes, and cardiovascular diseases has increased, and such diseases can be indicative of personal habits and physical activity [55]. Obesity and overweight are usually measured by using the body mass index (BMI) equation as follows.

(1) $$BMI=\frac{weight\left(kg\right)}{height^{2\;\left(m\right)}},$$

If the BMI is under 18.35, it means that the person is underweight. If the BMI is between 18.35 and 24.39, the condition is normal. If the BMI is between 25 and 29.39, it means that the person is overweight. If the BMI equals to or is more than 31, it means that the person suffers from obesity. The theorists in the field of urban design and travel behavior in urbanization and architecture began to study the placement pattern of uses, and local and transportation system design with respect to travel behavior in order to reduce traffic and climatic effects on life quality from 1980 [56,57]. During 1999, the WHO tried to relate urban design and planning with public health by focusing on the effect of individual behavior and relationship with human-made environments. Further, this report mentioned the aspects of urban environments affecting individual health through ecological dimensions, social networks, transportation, and residence [52]. A large number of studies were conducted on the relationship between natural, physical, and social environment and health during the last 10 years. In the early 21st century, the relationship between environmental tendencies and public health changed basically, and researchers reported a significant relationship between the human-made environment and health [58,59].

The above-mentioned literature highlights the importance of walking among physical activities. Walking is generally done for a special purpose and work, such as walking toward school and recreation in urban environments [60]. Physical activities in the city are divided into essential and working, recreational, and sports based on the theory of “Human City” by Gehl [61,62]. Regarding walkability, physical activity and behavior of pedestrians in the urban spaces of Sweden, Sundquist et al. [63]. (2011) reported a positive relationship between walking in urban environments and physical activity with respect to citizen health. Frank et al. assessed the relationship between urban environments and walking, representing that urban environment affects physical activity significantly by influencing walking, so that the individuals which avoid walking are likely to be overweight (2007) [64]. The results of BEPAS confirm that walking in Europe is directly related to physical activity and is associated with important economic advantages such as reducing costs [65]. Considering two distinct studies conducted on walkability in neighborhoods and physical activity in America and Australia, the results represent an effective relationship between the socioeconomic status of the environment, walking and physical activity [66,67,68]. Based on the report of the American surgical association (ASA), physical activity results in improving public health, and at least 31 min of daily walking is regarded as essential for individuals [19]. As previously mentioned, walking is the most important physical activity that can lead to the improvement of public health and physical fitness [69]. Considering spaces for physical activities along with the daily paths, would provide a comprehensive way of evaluating the citizen’s mobility as well as their spatiotemporal movement. A positive relationship between walking and the amount of physical activity is observed by considering the above-mentioned studies. Walking is conducted parallel to other activities and it is therefore a mean of mobility. Walking as a physical activity is associated with the least injuries. It is therefore has become a popular sport that attracts many and serves the main purpose of public health. Today, environmental design and planning should focus more on promoting walking as the most important factor of physical activity for improving public health [70]. Some urban designers and planners emphasize on the role of residential density, diversity, orientation, and design of pedestrian paths in urban spaces on individual health [71].

Accordingly, the physical features of urban spaces involve some factors and features related to human-made environments influencing the obesity, physical activity and nutrition of individuals [72]. Furthermore, the safety from vehicle traffic and security/safety from crime are features that are common in all components of the behavioral model of the environment and significantly correlate to the physical activity of individuals. Safety was defined as a comfort quality in some studies [73]. Motorized traffic volume, sidewalk width, path slope, spatial security, various uses, and night lighting are considered as some of the criteria affecting the quality of pedestrian presence in urban environments [66,74]. In addition, climatic conditions such as a very cold or warm climate, wind, and rain influence physical activities in urban environments [75]. Several researchers believe that the lack of barriers and differences in sidewalk levels is regarded as the reason for the importance of environmental criteria affecting the amount of walking and environmental health [76]. For instance, the distance of residence areas to the workplace, shopping spaces, recreational sports centers, the security of the environment, diversity of space users including female, male, children, youth and age, the volume of the visual information of environments such as the elements existing in space including building architecture (e.g., color and type of materials, decoration, style of architecture and flooring, building height), flooring, tree planting and green space, and the environmental factors such as a high slope of land, severe climatic factors such as severe radiation and wind, and width and quality of sidewalk [59,76,77,78,79,80,81,82,83], to note a few. Greater street connectivity has consistently been found associated with higher levels of walking [80]. Due to the availability of frequent commercial target places, a neighborhood with well-connected streets can be conducive to walking for logistics [84]. In practice, this may be the case of an incremental process, where economic activities and mobility would gradually advance around the well-connected streets [85]. Finally, it can be said that paying attention to citizens’ perceptions of their living environment is very useful in making the right decisions for urban planners who want to create a sustainable society [86]. Considering what was discussed above, it seems that, by increasing citizens’ daily physical activity, the probability of suffering from obesity and, subsequently, cardiovascular diseases, diabetes, and hypertension will decrease remarkably and the general health of the citizens will improve.

After extracting the four main criteria of urban morphology from the theoretical basis discussed above, each one of these criteria can be assessed with respect to urban planning and the physical design of urban spaces in order to obtain a set of sub-criteria for each one of them concerning urban planning. In Table 1, the criteria of urban morphology have been specified as the four main criteria. For each one of these criteria, some sub-criteria have been extracted with respect to the foundations of urban-space design from the perspective of physical design, hoping to design urban spaces and structures that can attract people. All four criteria of urban morphology, their design sub-criteria, and their descriptions can be found in Table 2.

## 3. Materials and Methods 

The criteria and the sub-criteria extracted from the urban morphology and the urban-space design literature were addressed in the questionnaires in order to determine the quantity of each physical activity. However, as spatial design could be incomprehensible and unexplainable to the public, and thus could not be directly incorporated in the questionnaire, its sub-criteria in the specified region were first analyzed by using space syntax in order to determine the linkage and connection in each urban space. Next, they were analyzed alongside other criteria with respect to the presence in each space. The criteria of urban morphology affecting physical activity and public health were grouped and prioritized through analytical network process (ANP) by using the questionnaire in order to evaluate the components of urban morphology provided in Table 2 and influence the public health of citizens. To this end, the physical-spatial, functional-activity, perceptual-visual and cultural-social criteria obtained by the questionnaires related to physical activity and walkability such as NEWS (Neighborhood Environment Walkability Scale), NPAQ (Neighborhood Physical Activity Questionnaire) and PAQ (Physical Activity Questionnaire) were tested and analyzed to determine each criterion and sub-criterion in the groups of physical activity and confirm the final effect of criteria in increasing physical activity. Accordingly, in the present study, for analyzing and weighting criteria, the questionnaires of two researchers and one expert are used. First the questionnaire was randomly and equally distributed among 360 males and females based on Cochran’s formula and the population using city structure daily, all of which were selected among space users as walking during the intended period (evenly and uniformly). Additionally, the second questionnaire was distributed among 20 experts and specialists in the field of health, physical activity, and urban design and morphology for weighting criteria through their pairwise comparisons (evenly and uniformly).

The criteria of urban morphology and their design sub-criteria, as mentioned in Table 1, were mixed, and the 12 criteria of urban morphology were obtained; that is, the criteria that were influential in the physical activity of the individuals and which could be used to design urban structures, environments and spaces. These criteria were later analyzed by using the following questionnaires.

The number of the required questionnaires was calculated by the Cochran formula with the sample size of 5500 (average daily citizen presence in the main structure of Hamadan City) and error percentage of 0.05. The 360 questionnaires were evenly distributed all over the main structure of the city for data collection. The questionnaire contained queries about each of the 12 specified criteria across four different categories of physical activity namely work, sports, recreation, and general. For example, the following question was about the criterion of “land use”:

To what extent does the variety of land use, such as commercial, sports and cultural, encourage you toward physical activity in the space?

A similar question was asked for each criterion and since four categories of physical activity were inquired about, the questionnaire totally consisted of 48 questions which the respondents answered using the 1–5 Likert scale. Based on the explanations on the statistical population and the sample population, Table 3 represents the questionnaire items and the percentage of answers given to each question in detail.

Additionally, considering the theoretical basis discussed in the present study, it is possible to reduce the level of obesity among the citizens to a great extent. In the distributed questionnaires, the weight and the height of the citizens were asked so that their BMI could be calculated and their relative obesity could be measured. Another question was about their medical records, including cardiovascular diseases, diabetes, high blood fat, and hypertension. These two variables could help us to assess the relationship between obesity and general health (cardiovascular diseases, diabetes, high blood fat, and hypertension). This relationship was assessed via the correlation test. In the first questionnaire, correlation analysis was performed. Those criteria whose correlation coefficient is more than 0.1 (whether positive or negative) and their significance level is less than 0.05, can affect the specific type of physical activity and their correlation can be significant. In the next step and the regression analysis, the minimum β must be more than 0.1; so that the specific criterion can be evaluated as affecting the related activity. Furthermore, R must be more than 0.1 so that R^2^ can help us to predict at least 1% of the physical activity. As mentioned previously, β can help us to determine the criteria affecting the physical activity and R and R^2^ can help us to determine the extent to which the criteria can affect the prediction of the physical activity. The effect of linkages and space syntax indexes on activities was analyzed and assessed by using depth map software. The relationship between human-made environments and walkability has been assessed from various perspectives and with various methods in numerous studies. A few of them are mentioned below. The relationship between human-made environments and walkability was studied with respect to the theory of space syntax and by using questionnaires. It was concluded that these two indicators had a positive correlation with one another [87,88]. Walkability in the environment was analyzed using GIS method and with respect to the four criteria; namely functional diversity, population density, business density, and intersection density. With the help of GIS, a raster data model was designed and the mentioned criteria were evaluated for obtaining the maps and quantities of each criterion. It was concluded that functional diversity plays a remarkable role in the walkability of neighborhoods [89,90,91]. Access is another environmental criterion that was analyzed by the GIS method. It was concluded that diverse access routes and targets can be greatly influential in encouraging people to walk [92]. 

The space syntax indexes were first examined in the main structure of the city and were then added to the general questionnaire according to the analysis of the syntax criterion to determine which spaces with the obtained indices people choose for each physical activity. They were evaluated using the ANP method, similar to other criteria including land use and climatic comfort whose individuality was determined.

Regarding the present study, the criteria of spatial integration, depth, connectivity were calculated and analyzed by this method. Integration as the most main concept in space syntax method is an axial map of the image related to spatial order of a city. The integration of a point in the map indicates its relationship with overall structure of the collection. Based on this definition, one space possesses more integration if it is possible to reach it by passing less space, and vice versa [51]. Regarding the present study, this method and depth map software were used to analyze and assess the indexes of integration, depth and connectivity which are regarded as the morphological criteria affecting the increase of the physical activity of citizens quantitatively and qualitatively as numerical and map, respectively. This criterion and its sub-criteria such as connectivity, depth and integration were analyzed and proved through space syntax method to obtain final criteria and sub-criteria influencing the increase of physical activity. Finally, the obtained and proved criteria were weighted through ANP to prioritize its criteria and sub-criteria for designing and implementing in specific temporal period. Since the 12 obtained variables were mutually correlated as well, they were compared and evaluated in pairs based on expert opinions using the ANP method so that their relationships could be taken into account in their prioritization.

### Analytic Network Process

The analytic network process was done by using the Super Decisions software. This software has been designed by the ANP team working in the Creative Decisions Organization [93].

ANP can be summarized in the following four steps [94]:

1. Creating a model and turning the issue into a network structure: In this step, the issue under investigation is turned into a network structure where knots are treated as clusters.
2. Creating a binary comparison matrix and specifying the priority vectors: similar to binary comparisons in AHP, decision components in each cluster are compared two by two, based on their importance with respect to control criteria. In the AHP method, each hierarchy has some clusters which can be weighted by using the matrix. However, in ANP, the components are divided into two parts: (a) the controller factor layer, and (b) the network layer. The first part consists of the decision’s criteria and goals (the main criteria), and the second part comprises all the components (the criteria and the sub-criteria) [95].
3. Creating a super matrix and turning it into a limit super matrix: In order to obtain the general priorities in an interactive system, the internal priority vectors (the calculated Ws) are inserted into the proper columns of a matrix. The result will be a super matrix (in fact, a divided matrix) in which, each part shows the relationship between two clusters in a system.
4. Choosing the best option: if the super matrix created in the third step covers the whole network, the options can be obtained from the normalized limit super matrix. If the super matrix covers only the interacting part of the network and the options are not considered in the super matrix, more calculations must be done to obtain the general priority of the options. The option with the highest general priority is chosen as the best option for the specific issue.

**Figure 2:**
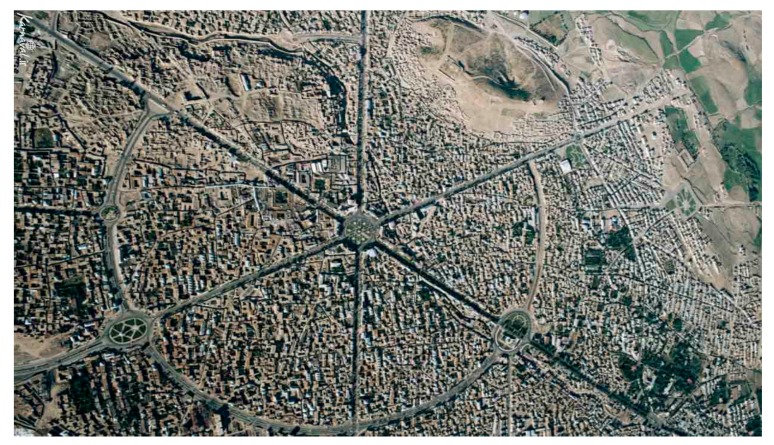
Radial form of the structure of Hamadan. Reference: Google Earth. 4 July, 2019.

As mentioned above, the central and main structure of Hamadan city in Iran, which is the radial structure including the central square of city and its six main streets, was selected as the case study of the present study due to its specific and radial morphology and structure and diversity of its spatial criteria. This structure is shown in Figure 2.

**Figure 3:**
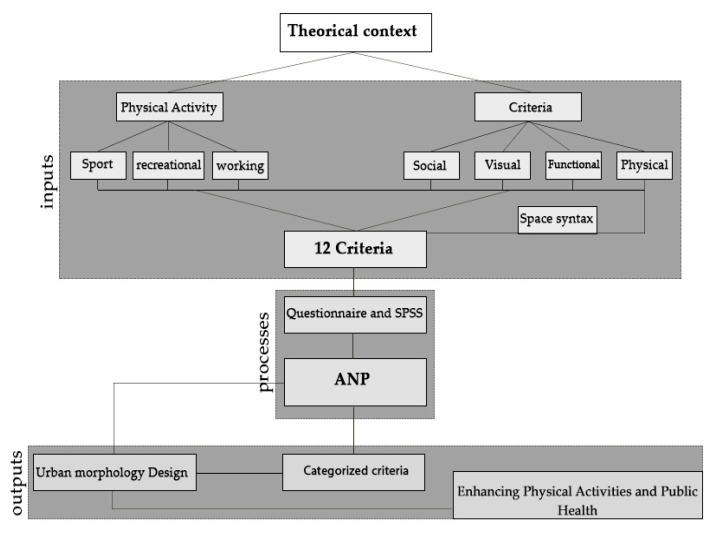
Different steps of the study.

According to Figure 3, four criteria for morphology and urban environment design, namely; (1) physical, (2) functional, (3) visual, and (4) sociocultural, and three types of physical activity, namely; (1) essential, (2) sport, and (3) recreational were determined as the research input. Via a review of the studies conducted in this field, 12 environmental design criteria that can affect physical activities were identified and were then processed and analyzed.

The processing consisted of two stages. In the first stage, the 12 input criteria were analyzed by a general questionnaire (answered by the public) in three categories of physical activity. Correlation and regression analyses were used for this purpose.

In the second stage, the four main criteria and their sub-criteria were processed by the ANP method for prioritization of the criteria in morphology and urban structure design. 

The outputs of these two stages were divided into two categories:

1. In the first stage (questionnaire), the criteria influential in each type of physical activity and the amount of their impact were determined.
2. In the second stage (ANP analysis), the criteria were prioritized, resulting in what is commonly known as classified criteria. This prioritization can be used in short-term and long-term planning in urban morphology and structure design.

The different stages of the study are schematically illustrated from beginning to end in Figure 3.

This research included two types of questionnaire. The initial questionnaire consisted of individual questions asked from the general population as described in Table 3. The data collected by this questionnaire were used for performing correlation and regression analysis and predicting the impact of each criterion on the different types of physical activity. The second questionnaire, which was used in the ANP method, inquired about the relationship between the criteria and the sub-criteria in pairs and individually by posing questions to 20 urban design experts and professors. The results were used to perform the ANP analyses in Super Decision software. Table 11 and Figure 8 show the coefficients and prioritization of the criteria. Urban morphology design means that urban planners and designers can use the above-said analyses and classified criteria obtained by questionnaire analysis and the ANP method to devise plans for designing urban morphology and structure over a specified period.

## 4. Results

**Figure 4:**
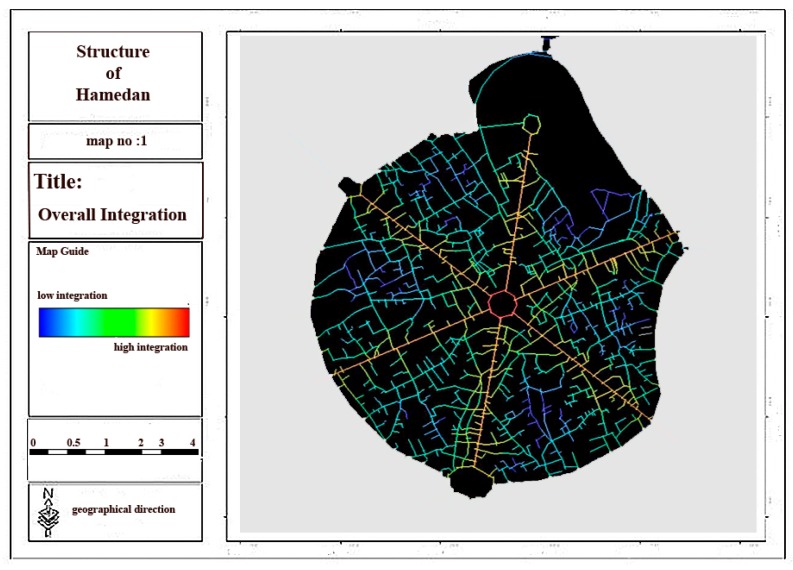
Overall integration of the main structure of city.

Regarding the analysis of relationship between spatial linkages and physical activities in urban spaces, space syntax indexes are regarded as one of the physical criteria of urban morphology by considering the above-mentioned theoretical basis. Space syntax method and UCL depth map software were used in this section to assess the effect of space syntax on the walking and physical activity of individuals in Hamadan city and its main structure in order to evaluate connectivity, integration and depth components as the main elements of urban structure and morphology. Then, considering the results obtained from the questionnaires and the interviews, the effects of space syntax and 11 other criteria on each physical activity were assessed. The ratio of the overall integration related to six main streets and central square of city to that of whole city was determined by considering the results and analyses conducted in depth map software (Figure 4).

**Figure 5:**
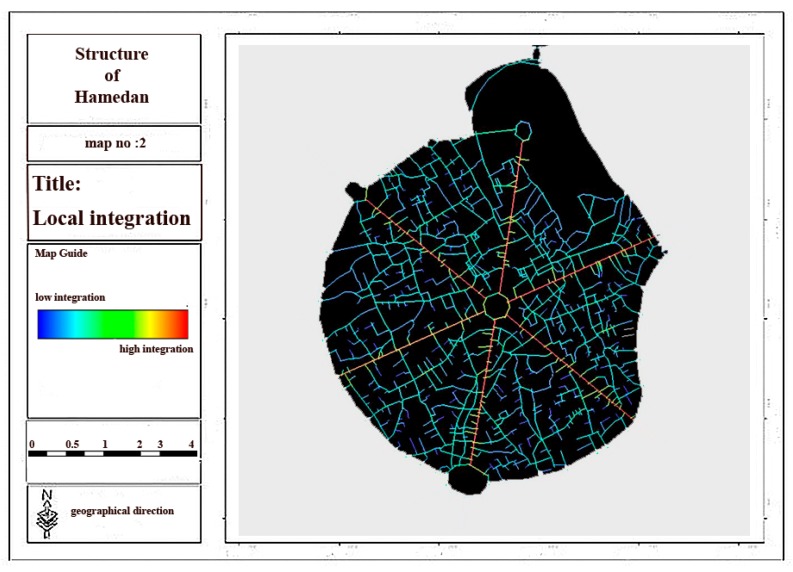
Local integration of the main structure of city.

**Figure 6:**
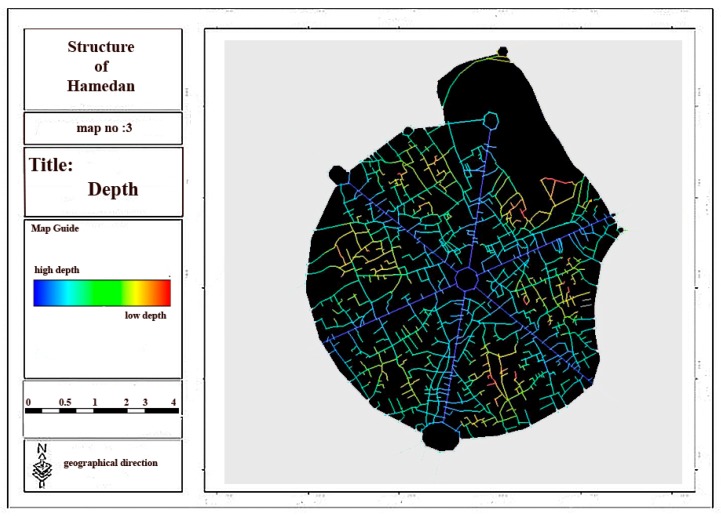
Depth of the main structure of city.

**Figure 7:**
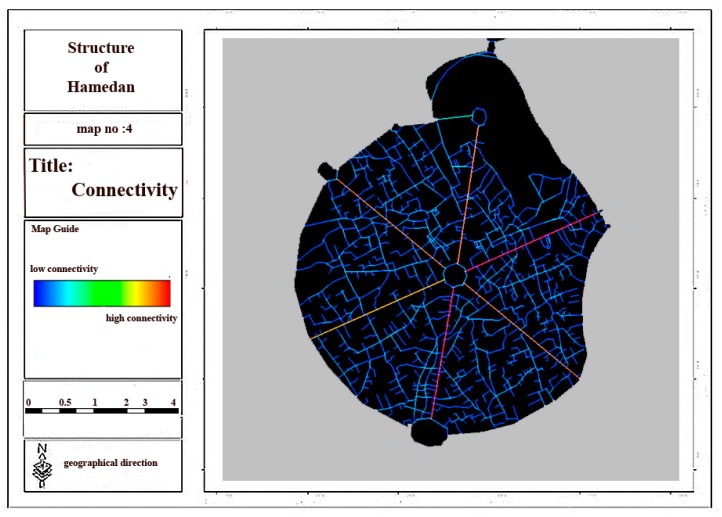
Connectivity in the main structure of city.

Considering Figure 4, Figure 5, Figure 6 and Figure 7 and Table 3, it can be concluded that the most local and overall integration can be seen in Bu-Ali Sina street and the least local and overall integration can be seen in Shohada street. Furthermore, the most connectivity and the least depth belong to Bu-Ali Sina street. The local and overall integration, the connectivity, and the depth of all six streets can be found in Table 4.

The criteria and sub-criteria related to urban morphology and public health, which were obtained from theoretical basis through a structural equation modelling approach, are assessed and analyzed in this section to determine and confirm the final criteria and sub-criteria influencing the increase in physical activity by using the questionnaire and their prioritization and weighting by using the ANP method. The relationship between the criteria of urban morphology and physical activity was assessed by using the correlation test and results are presented in Table 5.

The effective criteria and sub-criteria of urban morphology that can encourage citizens for daily walking in order to attain their purposes were specified by considering the correlation test, and each criteria and activity were analyzed separately. Based on the results, a positive relationship was observed between the variables of function, accessibility, climatic environmental comfort, and distance with purposeful, and working activity (Table 5). Furthermore, the variables of function, Accessibility, safety and security, climatic environmental comfort, vision and landscape (visual), and human scale were positively related with sports activity (Table 6). As shown in Table 7, a positive relationship was observed between the variables of function, space syntax indexes, climatic environmental comfort, urban facilities, infrastructure and furniture, vision and landscape, social and cultural, human scale, and memorable and historical texture with recreational activity. Additionally, the overall form and structure of the city is positively related to recreational activity. In general, the results represented a positive relationship between the variables of function, safety and security, climatic environmental comfort, space syntax indexes, facilities, infrastructure and furniture, vision and landscape, social and cultural, accessibility, human scale, memorable and historical texture, overall form and structure, and distance with physical activity. The variables affecting each one were entered into the equation by using a regression analysis test to predict working, sport, recreation, and general activity based on the criteria of urban morphology. The results of regression analysis test for predicting purposeful and working, sport, recreation, and general activity are summarized in Table 6, Table 7, Table 8 and Table 9.

According to Table 6, 5% of working activity could be predicted by the variables of function, transportation and climatic comfort together. Accordingly, function alone failed to predict working activity, while, accessibility (β = 0.16) and climatic comfort (β = 0.12) could influence essential and working activity. Further, the presence of higher accessibility and permeability (β = 0.16) and close distance (β = 0.22) maximally influenced essential and working activity based on the details of the results. The analysis of sport activity in Table 7 shows that the variables of function, accessibility, safety and security, climatic environmental comfort, vision and landscape and human scale alltogether could predict 7% of sports activity. In addition, sports activity could be predicted by function and accessibility with β = 0.17 and −0.17, respectively, while other entered variables failed to predict them.

Based on the results in Table 8, entered variables all together could predict 17% of recreational activity. Moreover, recreational activity could be predicted by function, space syntax indexes, facilities and infrastructure, overall form and structure, social and cultural issues, and memorable and historical texture with β 0.23, 0.25, 0.11, 0.21, 0.15, and 0.17, respectively. Regardless of the type of physical activity in general, questionnaire analyzes revealed that according to Table 9, the criteria of urban morphology could predict 18% of physical activity. Furthermore, physical activity could be predicted by the variables of function, safety and security, climatic comfort, space syntax indexes, overall form and structure, accessibility, distance, human scale, and historical texture with β of 0.16, 0.15, 0.11, 0.16, 0.12, 0.11, 0.13, 0.14, and 0.16, respectively. By designing the urban structure and morphology in accordance with the mentioned criteria, it is possible to enhance the citizens’ physical activities (including essential and working, sports, and recreational activities) up to 18%. The relationship between general health and obesity with respect to those listed in the materials and methods section using correlation analysis is shown in Table 10.

The results obtained from the questionnaires indicated that those who enjoyed general health and had no record of cardiovascular diseases, diabetes, high blood fat, and hypertension, were generally within the normal range of BMI and did not suffer overweight and obesity. However, the citizens with BMIs higher than the normal level mostly suffered from one of the above-mentioned diseases. The correlation test showed that there was a significant negative correlation between obesity and general health; that is, higher levels of obesity would increase the chance of catching one of the above-mentioned diseases and would lower the general health level. The results showed that the citizens with higher levels of obesity had lower levels of general health. Therefore, it can be concluded that by designing the urban structure and morphology in accordance with the design sub-criteria, it is possible to enhance physical activities up to 18% and by enhancing physical activities, it is possible to remarkably reduce the level of obesity, to reduce the chance of catching cardiovascular diseases, diabetes, high blood fat, and hypertension to a great extent, and consequently, to enhance the citizens’ general health. 

**Figure 8:**
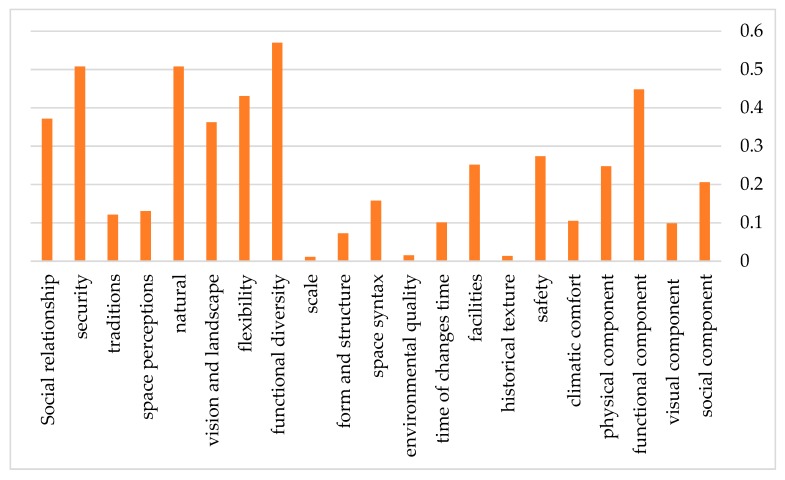
Criteria and sub-criteria weight.

The variables obtained from the urban morphology design were analyzed and compared based on the expert questionnaire as explained in the “Materials and Methods” section about the stages of the ANP method. Finally, they were scored using the Super Decisions software. Table 11 and Figure 8 show these scores.

**Figure 9:**
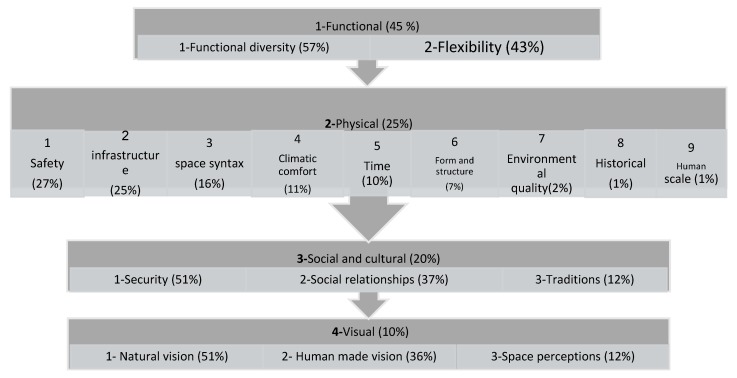
Prioritizing the criteria and the sub-criteria affecting physical activities.

Considering the analysis of the obtained results and the information presented in Table 10 and Figure 7 and Figure 9, it was concluded that among the main criteria of urban morphology, the functional component with 45% could be the most effective criterion in the structural design of urban spaces. The physical component, the social component, and the visual component took the next positions with 25%, 20%, and 10%, respectively. The sub-criteria of each one of these criteria which can help us design the urban structure, are arranged according to their position and effectiveness level and can be found in Figure 9.

## 5. Discussions

In the late 19th century, following the spread of contagious diseases, the role of urban design and planning in the physical activities of users was recognized as a significant factor in enhancing general health [11]. Nowadays, urban design and planning is known as an important factor in enhancing the health of citizens and encouraging them to engage in physical activities, which are considered as the first priorities in enhancing the health of urban societies. Previous studies show that inattention to urban morphology, functional diversity, urban facilities and furniture, urban pedestrian space, etc., are directly related to physical inactivity, overweight, obesity and mental health problems in the society [96]. Thus, in the recent approaches to urban design and planning, numerous studies put emphasis on achieving healthy, efficient, flexible, and sustainable cities. Considering the low success of individual attitudes and suggestions aiming at improving the health of urban societies, nowadays, social and ecological approaches and attitudes to urban design and planning in areas such as walkability, sports activities, and physical activities, are in the spotlight [97]. Bentley et al. [98] believe that today, healthcare experts and policy-makers prefer to turn to ecological approaches to general health. Social and ecological attitudes emphasize the role of human-made and artificial factors in enhancing physical activities and consequently, urban health [99,100,101]. Paying attention to the various dimensions of environmental design in open urban spaces can lead to paying attention to walkability, neighborhood-based planning, public transportation, the environment and the distribution of public and green spaces, and consequently, leading to a comprehensive and united approach to enhancing physical activities and urban health [102].

Although there is rich literature related to the studies conducted on the environmental factors affecting physical activities, few empirical studies have been conducted in this area and most of the studies in this area have been qualitative. This study comprehensively and systematically reviewed the role of urban morphology design in enhancing physical activities and consequently, reducing the level of obesity and the probability of catching cardiovascular diseases, diabetes, high blood fat, and hypertension in urban spaces, and confirmed the existence of a relationship between the quality of urban design and physical activity. Furthermore, the criteria of urban morphology design positively affecting physical activities were quantitatively analyzed, so that the effect of each criterion and its level of priority could be precisely and numerically determined. In this study, based on the analysis of deductive statistics, including correlation coefficient, regression, and binary analysis, and with the use of the data obtained from the questionnaires distributed among 360 users of the urban spaces, the environmental factors affecting physical activities were classified into 4 criteria, namely physical, functional, visual, and social, and 11 sub-criteria. These criteria and sub-criteria were analyzed in three different dimensions, namely working and essential activities, sports activities, and recreational activities—this can be regarded as one of the special and outstanding features of the present study.

From the physical criteria affecting physical activities, spatial safety, accessibility, urban facilities and furniture, spatial integration and connectivity, and environmental comfort were the most important priorities obtained from the analyses. Most of the previous studies have emphasized the existence of a direct relationship between these criteria and physical activities [66,67,68,69,70,71,72,73]. From the functional criteria, functional diversity and spatial flexibility played a significant role in enhancing physical activities. Although the effect of functional criteria on physical activities and urban health was not proved to be definite and clear, most researchers have acknowledged the role of functional diversity in urban spaces and its relationship with physical activities, and specifically walking in urban spaces [103]. Furthermore, considering the functional criteria, factors such as access to public parking spaces, the existence of retailers, and spatial contingency were also emphasized with respect to enhancing physical activities. For example, the accessibility of diverse and attractive spaces such as local stores and services has been introduced as one of the most influential factors in walkability [104]. Concerning the perceptual and ecological criteria, the role of urban visions and environmental landscapes, such as the quality of walls, urban facades, and skylines [62], and concerning natural visions and landscapes, the role of trees, and green and natural spaces and elements in enhancing the physical activities and the tendency to walk, to exercise, and to go shopping should be mentioned [105,106]. From among the social and cultural criteria, social security in spaces and social interactions and relationships were the most important factors in regard to the citizens’ presence in urban spaces and enhancing physical activities in urban spaces. In regard to the social criteria of urban spaces influential in enhancing physical activities, some of the most significant factors and indicators have been as follows: attachment to a place, the existence of memorable texture in public spaces, the history of a place, and social relationships [107,108]. However, there are also few studies showing that there is no relationship between spatial security and physical activity [109]. 

The researchers also confronted some limitations while assessing the role of urban morphology design and environmental factors in enhancing physical activities and urban health. One of these limitations was the lack of precise theories and empirical evidence. Very few studies have put aside generalizations about physical activity and urban design in favor of providing a more precise categorization of the criteria that affect each area of urban design. Another challenge that researchers in this area may confront is the limitation in employing functional methods and techniques of assessing physical activities affected by environmental factors. Most methods in this field are qualitative and descriptive, and consequently have limited use for verifiably quantifying the significance and priority of each influential criterion [110].

One of the limitations of this project was the measurement of the statistical population. No accurate statistics were available for correct estimation of the daily presence of people in the main structure of the city and unofficial statistics were not accurate or reliable. Another limitation was related to the application of a proper method for obtaining the classified criteria. There were two options available to the authors: the GIS platform and ANP analysis. Since accurate GIS data and maps were not yet available at the time of conducting this research, the authors had to settle for ANP analysis; however, it was very difficult to find 20 experts of urban structure and morphology design in Hamadan who were knowledgeable about the mentioned criteria. There were also some limitations in the theoretical foundations section—especially in morphology discussions—since some scholars limit urban morphology to the physical form and body of cities while others incorporate socioeconomic concepts into it. Therefore, multiple definitions of urban morphology had to be presented and coalesced into an acceptable frame.

## 6. Conclusions

Physical activity and health are among the most significant issues in the area of urban design and planning. Based on the analyses performed in this study, four dimensions of environmental quality—namely, physical, functional, visual, and social quality—were among the most influential factors in enhancing physical activities, including working, sports, and recreational activities, in urban open spaces. Examining the criteria and the sub-criteria of designing environmental components of urban morphology showed that factors such as functional diversity, accessibility, climatic comfort, and the distance from the working activity were among the most important environmental features affecting working activities. Regarding recreational activities, environmental factors such as functional diversity, climatic comfort, urban facilities and furniture, natural and artificial visions and landscapes, the social and cultural features of the environment, the human scale in space, and the historical identity of the space played a significant role. Regarding sports activities, the most influential environmental factors were the functional diversity of the space, safety and security, climatic comfort, visions and landscapes (visual), and the human scale in open urban spaces.

Furthermore, according to Table 10, spatial security, accessibility and urban facilities were among the physical criteria most greatly affecting physical activities. From among the functional criteria, functional diversity of the space and spatial flexibility were the most influential factors in enhancing physical activities. Concerning the perceptual criteria, the role of urban visions and artificial landscapes, such as the quality of walls, urban facades, and skylines, and concerning natural visions and landscapes, the role of trees, and green and natural spaces in enhancing physical activities, were remarkable. Finally, from among the social and cultural criteria, social security in spaces and social interactions and relationships were the most important factors in regard to the citizens’ presence in urban spaces and enhancing physical activities in urban spaces. Considering the BMI of those who filled the questionnaires and their health level with respect to diseases attributable to obesity and physical inactivity (cardiovascular diseases, diabetes, high blood fat, and hypertension), it was found out that there was a very significant correlation between obesity and the probability of catching these diseases. It was also found out that urban morphology design could lead to an increase in the walking activity and other activities of citizens, and consequently, to the reduction of the obesity level, the reduction of the probability of catching the above-mentioned diseases as well as the improvement of citizens’ general health level. 

Based on the results of statistical and software analyses, urban structure and morphology directly influence the increasing presence and physical activity of citizens and improve the mean of public health level. Urban morphology, as the first and important component of a city, can play a major and fundamental role in attracting pedestrian population and improving public health in the community. The theoretical studies represents that physical activity within urban environment is divided into working, recreational, and sports, and the morphological criteria affecting each of these activities in the field of urban planning and designing are classified into physical–spatial, functional–activity, cultural–social, and perceptual–visual groups. A positive relationship was observed between the variables of function, quality, access distance and climatic-environmental comfort with purposeful and working activities in urban spaces by considering the analytical results obtained from inferential statistics and spatial analyses of space syntax indexes. Moreover, the variables of function, safety and security, climatic environmental comfort, visual vision and landscape, human scale were positively related to sports activity. Furthermore, there was a positive relationship between the variables of function, climatic environmental comfort, facilities and infrastructure, quality of urban furniture, vision and landscape, social and cultural features, human scale, and memorable and historical identity of urban spaces with recreational activity.

Functional diversity (mixing various 24-h uses) and spatial flexibility are regarded as the main criterions to be considered when designing urban spaces for increasing the level of daily physical activity. Regarding the physical criterion obtained as one of the important criteria affecting physical activity, paying attention to spatial safety, climatic comfort and spatial integration as physical-spatial sub-criteria play an important role on improving physical activities and urban health. Social security, and the presence of various social groups play an important role in enhancing the public health of community by concerning cultural-social criteria. Finally, paying attention to natural vision and landscape such as trees, water, and green space and artificial cases such as creating beautiful walls, flooring, and landscaping, are regarded as important by considering the perceptual–visual criteria. Thus, it is concluded that the collaboration and the establishment of an interdisciplinary link between urban planning and the healthcare system can be influential in enhancing the environmental features of urban spaces and encourage individuals to attend urban spaces and, thus, be decisive in enhancing urban health. The health-oriented approach to urban design and planning can improve the healthcare system in the societies of today. It is possible to make scheduled and consistent plans for designing urban structures and spaces in accordance with the priorities and the criteria discussed in the present study. Considering the economic, social, etc., limitations, this can help us to gradually execute urban plans and, subsequently, to enhance the physical activities and general health of the citizens.

## Figures and Tables

**Table 1:** Conceptual dimensions of morphology according to different theorists.

	Theorist	Concepts	Common Point
**Foreign**	Cortes	Form, city structure, proportions and deformation of objects and their components	Theorists mostly emphasize on the physical-functional aspects of city such as street and building pattern, which urban designers can interfere in its formation. Further, social, economic and political factors and the passing of time significantly affect the formation of the artifact texture of the city.
	Moudon	Economic and social structures, time and its effect on city form	
	Kropf	Use of buildings, and human activities and interactions	
	Jones and Larkham	Form, cultural factors, and urban components and landscape	
	Roofeh	Historical and social factors, form and spatial factors	
	Whighthand	Physical form of the city	
	Kermona	Form and space	
	Sheer	Form and time	
	Scholz	Implementation, extension, and spatial interaction	
	Guitergrind	Form and shape	
**Local**	Mir Moghtadaei	Form, shape and city structure	
	Madani Pour	Form, shape, structure and operation of artifact urban texture	

**Table 2:** Criterion and sub-criterion of urban morphology design affecting an increase in physical activity.

Criterion	Sub-Criterion of Urban Design	Index	Description
**Physical and spatial**	Overall form and structure of city	Street layout	Passing form of street involving straight, curved, and broken paths
		Land subdivision	Initial pattern of land subdivisions and buildings that create texture form
		Density	Density of the existing buildings in the main structure of the city
		Using the different patterns of urban block	Overall form and shape of the city structure including checkered, radial and linear
		Distance between origin and destination paths	Distance between two main urban spaces
		Full and empty space (open and close space)	Ratio between open, closed, and semi-open spaces
		Hierarchy Index	
	Safety	pedestrian and rider	Pedestrian safety against passing rider
		Environmental	Rider and cyclist safety against natural disasters
	Environmental quality	Land slope	Slope of passing path proportional to pedestrian and cyclist
		Materials	Visual and functional quality of used materials
		Proportions	Proportions used in urban spaces
	Urban facilities and infrastructure	Urban furniture	Furniture used in urban spaces, including sitting area, electric lamp, waste basket, areas for drinking water, tree, canopy, traffic signs, etc.
		Flooring	Flooring type used in the passing paths of pedestrian and cyclist
		Surface and waste-water disposal	Method of disposing the wastewater and surface waters existing in passing paths
		Pedestrian crossing	Facilities for separating rider and pedestrian
	Scale	Enclosure Index	
		Human scale	Proportion between urban walls and spaces with human feelings
		Skyline	Terminating the line of the buildings existing in the space
	Climatic comfort	Physical	Comfort versus atmospheric conditions by using porch and canopy
		Natural	Comfort versus atmospheric conditions by using tree, water, etc.
	Historical and memorable texture	Valuable historical building	Memorable and historical single-building existing in urban structure
		Ancient historical texture and wall	Ancient wall and valuable texture
	Time	Physical changes	Short- and long term periodic changes in natural and artificial environments, leading to the physical change, development and instruction of elements over time
	Space syntax indexes	Connectivity, accessibility and permeability	Number of the paths which are directly related to the path under analysis
		Depth	Minimum spatial step between one node or path to other node or path
		Integration	Mean number of lines from which all lines and routes can be accessed
**Function and activity**	Functional diversity	Commercial use	Existence of various commercial uses
		Cultural and social use	Existence of various cultural social uses
		24-h use	Existence of long-lasting and 24-h uses
		Street use	Permeating uses into space
		Human activities	Existence of human and human activities such as social interaction, walking, and shopping in space
	Flexibility	Use	Flexibility, variability and diversity of uses, human activities and physical space under use for diverse and 24-h efficiency
		Activities	
		Physical	
**Visual and perceptional**	Artificial vision and landscape	Wall	Improving the factors related to vision in human-made landscapes in passages, attractive and varied views, and presence of absorbing urban elements and signs
		Sigle-building	
		Urban signs	
	Natural vision and landscape	Greenness	Enhancing the factors related to vision to natural landscape in passages, green spaces and diversity of vegetation, and vision in urban landscapes and environment
		Water	
		Landscape of environment	
	Spatial perceptions	Mind map	Improving the mental properties related to physical activity and perceptual features of the city
		Legibility	
**Social and cultural**	Traditions	Demonstration	Civil, religious and cultural-political activities and communities within city structure
		Ritual and religious ceremonies	
		Sports march	
	Security	Social monitoring	Promoting the factors related to social monitoring, active and bright points at night, furniture and lighting
		Lighting	
		Active points	
	Social relationships	Active environment	Designing the public and civil spaces of city which are considered as an arena of appearing the civil and social thoughts and interactions of citizens and leads to the social health and happiness of citizens, along with help to administer community through attracting their public participations
		Cluster environment	
		Street use	

**Table 3:** General questionnaire questions and answers.

Variable	Categories	Percentage (%)			
		Types of Physical Activity			
		Essential and Working	Sports	Recreational	Overall
Age	13–18	6.7	6.7	6.7	6.7
	19–25	26.7	26.7	26.7	26.7
	26–40	30.0	30.0	30.0	30.0
	41–60	26.7	26.7	26.7	26.7
	61–75	10.3	10.3	10.3	10.3
Gender	Men	52.4	52.4	52.4	52.4
	Women	47.6	47.6	47.6	47.6
Weight	40–55	16.11	16.11	16.11	16.11
	56–70	21.11	21.11	21.11	21.11
	71–90	26.38	26.38	26.38	26.38
	91–110	18.33	18.33	18.33	18.33
	+110	18.05	18.05	18.05	18.05
Height	150–160	12.50	12.50	12.50	12.50
	160–170	23.05	23.05	23.05	23.05
	170–180	28.33	28.33	28.33	28.33
	180–190	21.94	21.94	21.94	21.94
	+190	14.16	14.16	14.16	14.16
Disease	Diabetes	7.22	7.22	7.22	7.22
	Cardiovascular	10.27	10.27	10.27	10.27
	Hypertension	10.55	10.55	10.55	10.55
	Blood fat	9.44	9.44	9.44	9.44
	None	62.50	62.50	62.50	62.50
Function	Very low	27.50	23.50	27.62	27.77
	Low	6.11	17.55	4.11	3.91
	Medium	21.11	13.45	22.23	20.88
	High	24.16	21.23	15.03	18
	Very High	21.11	24.27	31.01	29.44
Accessibility	Very low	20.30	22.90	23.20	21.34
	Low	20.65	19.71	19.85	19.65
	Medium	14.02	10.13	10.41	13.59
	High	24.25	24.07	24.26	24.16
	Very High	20.78	23.19	22.28	21.26
Distance	Very low	23.33	25.00	25.27	23.61
	Low	19.44	19.16	18.88	11.11
	Medium	9.44	8.61	8.88	27.50
	High	23.33	23.61	23.33	6.11
	Very High	24.44	23.61	23.61	31.66
Safety and security	Very low	26.38	26.11	25.00	25.83
	Low	11.38	11.11	14.16	11.11
	Medium	25.00	24.72	21.11	24.72
	High	6.66	4.44	11.94	4.44
	Very High	30.55	33.61	27.77	33.88
Climatic environmental comfort	Very low	23.33	23.05	25.55	25.27
	Low	19.44	19.72	14.16	13.33
	Medium	9.44	10.00	17.77	18.05
	High	23.33	23.88	11.94	11.38
	Very High	24.44	23.33	30.55	31.94
Space syntax indexes	Very low	26.38	28.05	24.72	25.55
	Low	11.38	13.33	13.61	11.11
	Medium	25.00	17.77	18.05	15.55
	High	6.66	11.38	11.94	12.77
	Very High	30.55	29.44	31.66	35.00
Facilities, infrastructure and furniture	Very low	25.00	25.00	26.94	27.50
	Low	19.16	19.16	10.00	6.11
	Medium	8.61	8.61	15.55	21.11
	High	23.61	23.61	20.27	24.16
	Very High	23.61	23.61	27.22	21.11
Overall form and structure	Very low	28.05	28.33	21.34	23.33
	Low	6.66	6.94	19.65	19.44
	Medium	21.38	21.38	13.59	9.44
	High	27.50	27.77	24.16	23.33
	Very High	16.38	15.55	21.26	24.44
Human scale	Very low	27.50	27.50	22.50	26.11
	Low	7.22	6.66	16.11	16.66
	Medium	21.66	17.50	6.11	6.66
	High	28.05	26.94	24.44	21.66
	Very High	15.55	21.38	30.83	28.88
Memorable and historical texture	Very low	28.05	27.77	18.88	23.05
	Low	13.33	16.66	10.00	19.72
	Medium	17.77	6.66	8.05	10.00
	High	11.38	21.94	25.83	23.88
	Very High	29.44	26.94	37.22	23.33
Natural and artificial vision and landscape	Very low	30.55	27.50	22.22	30.00
	Low	12.77	6.11	12.50	12.22
	Medium	7.50	21.11	5.27	5.00
	High	18.61	24.16	21.11	21.66
	Very High	30.55	21.11	38.88	31.11
Social and cultural	Very low	6.94	25.00	30.00	30.83
	Low	33.33	19.16	11.66	11.94
	Medium	27.77	8.61	5.00	4.72
	High	13.88	23.61	20.55	21.38
	Very High	18.05	23.61	32.77	31.11

**Table 4:** Comparison of space syntax indexes in urban structure.

Overall Integration	Local Integration	Connectivity	Depth
0.978	3.91	28	9.33
0.960	3.72	24	9.53
0.931	3.31	21	9.80
0.975	3.83	27	9.40
0.947	3.58	25	9.65
0.975	3.51	25	9.40

**Table 5:** Correlation coefficients between the criteria of urban morphology and physical activities.

Activity Sub Criterion	Essential and Working		Sports		Recreational		Overall	
	Correlation	Sig.	Correlation	Sig.	Correlation	Sig.	Correlation	Sig.
**Function**	0.10	0.04	0.18	0.001	0.26	0.001	0.27	0.001
**Accessibility**	0.19	0.001	0.13	0.01	0.07	0.16	0.17	0.16
**Distance**	0.15	0.001	0.03	0.56	0.02	0.002	0.16	0.001
**Safety and security**	0.05	0.30	0.11	0.04	0.07	0.16	0.12	0.03
**Climatic comfort**	0.15	0.005	0.12	0.02	0.15	0.005	0.21	0.001
**Space syntax indexes**	0.05	0.002	0.01	0.001	0.23	0.003	0.32	0.001
**Facilities, infrastructure, and furniture**	0.03	0.57	0.03	0.057	0.23	0.01	0.10	0.04
**Overall form and structure**	−0.04	0.44	−0.08	0.12	0.17	0.001	0.15	0.004
**Human scale**	−0.05	0.002	0.14	0.01	0.42	0.001	0.19	0.002
**Memorable and historical texture**	0.01	0.001	0.06	0.12	0.69	0.001	0.12	0.01
**Natural and artificial vision, and landscape**	0.08	0.14	0.10	0.04	0.52	0.006	0.16	0.002
**Social and cultural**	0.04	0.49	0.03	0.59	0.19	0.001	0.13	0.01

**Table 6:** Results of regression analysis for predicting essential and working activity.

Criterion Variable	R	R^2^	F	Sig.	Predicting Variable	B	β	T	Sig.
**Working Activity**	0.23	0.05	6.92	0.001	Function	0.06	0.07	1.37	0.17
					Accessibility	0.32	0.16	3.13	0.002
					Climatic environmental comfort	0.07	0.12	2.22	0.03
					Distance	0.29	0.22	2.89	0.003

**Table 7:** Results of regression analysis test for predicting sports activity.

Criterion Variable	R	R^2^	F	Sig.	Predicting Variable	B	β	T	Sig.
**Sports Activity**	0.27	0.07	5.76	0.001	Function	0.14	0.17	3.32	0.001
					Accessibility	−0.32	−0.17	3.32	0.001
					Safety and security	0.03	0.05	0.89	0.37
					Climatic environmental comfort	0.06	0.09	1.65	0.10
					Human scale	0.12	0.03	2.22	0.002
					Vision and landscape	0.06	0.07	1.26	0.21

**Table 8:** Results of regression analysis test for predicting recreational activity.

Criterion Variable	R	R^2^	F	Sig.	Predicting Variable	B	β	t	Sig.
**Recreational activity**	0.42	0.17	11.92	0.002	Function	0.21	0.23	4.74	0.001
					Space syntax indexes	0.22	0.25	5.22	0.001
					Climatic environmental comfort	0.07	0.10	1.86	0.06
					Facilities, infrastructure, and furniture	0.14	0.11	2.19	0.03
					Overall form and structure	0.40	0.21	4.35	0.001
					Human scale	0.22	0.04	3.32	0.06
					Memorable and historical texture	0.18	0.17	1.25	0.002
					Vision and landscape	0.09	0.09	1.68	0.09
					Social and cultural	0.21	0.15	3.07	0.002

**Table 9:** Results of regression analysis test for predicting physical activity.

Criterion Variable	R	R^2^	F	Sig.	Predicting Variable	B	β	T	Sig.
**Physical activity**	0.43	0.18	10.54	0.001	Function	0.41	0.16	4.88	0.001
					Safety and security	0.19	0.15	2.26	0.02
					Climatic environmental comfort	0.18	0.11	2.56	0.01
					Space syntax indexes	0.22	0.16	3.22	0.001
					Facilities, infrastructure, and furniture	0.13	0.05	1.04	0.30
					Overall form and structure	0.86	0.12	4.56	0.001
					Vision and landscape	0.15	0.07	1.51	0.13
					Social and cultural	0.26	0.09	1.77	0.08
					Human scale	0.22	0.14	4.44	0.001
					Memorable and historical texture	0.18	0.16	3.23	0.002
					Accessibility	0.25	0.11	3.55	0.001
					Distance	0.38	0.13	4.12	0.002

**Table 10:** Correlation coefficients between obesity and public health.

	Public Health (Diabetes, Cardiovascular, Blood Fat, Blood Pressure)	
**BMI (Obesity)**	Correlation	Sig.
	0.65	0.01

**Table 11:** Weighted super matrix and the limit of criteria and sub-criteria.

Variables	Name	Normalized by Cluster	Limiting
Main criteria	Social and cultural component	0.205	0.079744
	Visual component	0.098	0.03832
	Functional component	0.447	0.17366
	Physical component	0.247	0.095945
Physical	Climatic comfort	0.104	0.021682
	Safety	0.273	0.056533
	Historical texture	0.013	0.002823
	Facilities, infrastructure, and furniture	0.251	0.051922
	Time of changes	0.101	0.020913
	Environmental quality	0.015	0.003102
	Space syntax indexes	0.157	0.032529
	Form and structure	0.072	0.01497
	Human scale and skyline	0.010	0.002271
Functional	Functional diversity	0.569	0.1513
	Flexibility	0.430	0.114241
Visual	Human-made vision and landscape	0.362	0.015955
	Natural	0.507	0.022365
	Space perceptions	0.130	0.005749
Social	Traditions	0.121	0.011624
	Security	0.507	0.048693
	Social relationships	0.371	0.035657
